# Supplementary material for: Validation of non-participation bias methodology based on record-linked Finnish register-based health survey data: a protocol paper
Source: BMJ Open. 2019 Apr 4;9(4):e026187. doi: 10.1136/bmjopen-2018-026187 (PMC6500270; doi:10.1136/bmjopen-2018-026187)
Supplement: Author's manuscript [file bmjopen-2018-026187.draft_revisions.pdf]

BMJ Open

BMJ Open is committed to open peer review. As part of this commitment we make the peer review history of every article we publish publicly available.

When an article is published we post the peer reviewers' comments and the authors' responses online. We also post the versions of the paper that were used during peer review. These are the versions that the peer review comments apply to.

The versions of the paper that follow are the versions that were submitted during the peer review process. They are not the versions of record or the final published versions. They should not be cited or distributed as the published version of this manuscript.

BMJ Open is an open access journal and the full, final, typeset and author-corrected version of record of the manuscript is available on our site with no access controls, subscription charges or pay-per-view fees (http://bmjopen.bmj.com).

If you have any questions on BMJ Open's open peer review process please email info.bmjopen@bmj.com

BMJ Open

Validation of non-participation bias methodology based on record-linked Finnish register-based health survey data: a protocol paper

Journal:	*BMJ Open*
Manuscript ID	bmjopen-2018-026187
Article Type:	Protocol
Date Submitted by the Author:	21-Aug-2018
Complete List of Authors:	McMinn, Megan; University of Glasgow, MRC/CSO Social and Public Health Sciences Unit<br>Martikainen, Pekka; University of Helsinki, Faculty of Social Science, Population Research Unit<br>Gorman, Emma; Lancaster University, Department of Economics<br>Rissanen, Harri; National Institute for Health and Welfare, Department of Public Health Solutions<br>Härkänen, Tommi; National Institute for Health and Welfare, Department of Public Health Solutions<br>Tolonen, Hanna; National Institute for Health and Welfare , Department of Public Health Solutions<br>Leyland, Alastair; University of Glasgow, MRC/CSO Social and Public Health Sciences Unit<br>Gray, Linsay; University of Glasgow, MRC/CSO Social and Public Health Sciences Unit
Keywords:	Protocols & guidelines < HEALTH SERVICES ADMINISTRATION & MANAGEMENT, PUBLIC HEALTH, STATISTICS & RESEARCH METHODS

SCHOLARONE™
Manuscripts

Validation of non-participation bias methodology based on record-linked Finnish register-based health survey data: a protocol paper

Megan A. McMinn, MRC/CSO Social and Public Health Sciences Unit, University of Glasgow, 200 Renfield Street, Glasgow, UK

Pekka Martikainen, Population Research Unit, Faculty of Social Science, University of Helsinki, Finland

Emma Gorman, Department of Economics, Lancaster University, Bailrigg, Lancaster, UK

Harri Rissanen, Public Health Solutions, National Institute for Health and Welfare (THL), Mannerheimintie 166, Helsinki, Finland

Tommi Härkänen, Public Health Solutions, National Institute for Health and Welfare (THL), Mannerheimintie 166, Helsinki, Finland

Hanna Tolonen, Public Health Solutions, National Institute for Health and Welfare (THL), Mannerheimintie 166, Helsinki, Finland

Alastair H. Leyland, MRC/CSO Social and Public Health Sciences Unit, University of Glasgow, 200 Renfield Street, Glasgow, UK

Linsay Gray, MRC/CSO Social and Public Health Sciences Unit, University of Glasgow, 200 Renfield Street, Glasgow, UK

Corresponding author: Megan McMinn

MRC/CSO Social and Public Health Sciences Unit

200 Renfield Street

Glasgow, G2 3AX

megan.mcminn@glasgow.ac.uk

Tel: 0141 535 7643

Word count: 2,409

For peer review only

ABSTRACT

Introduction

Decreasing participation levels in health surveys pose a threat to the validity of estimates intended to be representative of their target population. If participants and non-participants differ systematically, the results may be biased. The application of traditional non-response adjustment methods, such as weighting, can fail to correct for such biases, as estimates are typically based on the sociodemographic information available. Therefore, a dedicated methodology to infer on non-participants offers advancement by employing survey data linked to administrative health records, with reference to data on the general population. We aim to validate such a methodology in a register-based setting, where individual-level data on participants and non-participants are available, taking alcohol consumption estimation as the exemplar focus.

Methods and analysis

We make use of the selected sample of the Health 2000 survey conducted in Finland, and a separate 11% register-based sample of the contemporaneous population, with follow-up until 2012. Finland has nationally representative administrative and health registers available for individual level record-linkage to the Health 2000 survey participants and invited non-participants, and the 11% population sample. By comparing the population sample and the participants, synthetic observations representing the non-participants may be generated, as per the developed methodology. We can compare the distribution of the synthetic non-participants with the true distribution from the register data. Multiple imputation is then used to estimate alcohol consumption based on both the actual and synthetic data for non-participants, and the estimates can be compared to evaluate the methodology's performance.

Ethics and dissemination

Ethical approval and access to the Health 2000 survey data, and data from administrative and health registers has been given by the Health 2000 Scientific Advisory Board, Statistics Finland and the National Institute for Health and Welfare. The outputs will include two publications in public health and statistical methodology journals, and conference presentations.

For peer review only

ARTICLE SUMMARY

Strengths and Limitations of this study

This study will validate a dedicated methodology which aims to adjust for non-participation bias in health surveys through the use of record linkage.

We use an individual level dataset on the entire selected sample for a Finnish national health survey, from which the characteristics of non-participants can be identified, with linkage to morbidity and mortality records, providing the "gold standard" for the methodology validation process.

Previous applications of this methodology have been able to use data on the total population for comparison, this study is limited to the 11% population sample available for this analysis.

The estimated gradient in the risk of alcohol-related harms may be stronger using individual measures of socioeconomic position than area level measures of deprivation; therefore these reference comparisons may not mirror the methodology based on less informative area-based measures.

This validation exercise is confined to assessing the reliability of inferring on non-participants from comparisons of the participants and the reference population; other aspects of the methodology such as the extent to which alcohol-related hospitalisations and deaths provide sufficient information to impute unknown alcohol consumption estimates are beyond the scope of this study.

For peer review only

INTRODUCTION

Health surveys enable the production of estimates of various health-related behaviours, such as smoking prevalence, levels of physical activity and alcohol consumption for entire populations, not confined to the sub-population in contact with health services. However the decreasing levels of participation in these surveys threaten their ability to provide reliable estimates.[1-3] The proportions of non-participation are typically not uniform across sociodemographic groups, meaning that selected groups, such as men or those from deprived backgrounds, are often underrepresented in health surveys.[4] Non-participation has also been found to correlate with higher rates of morbidity and mortality;[5, 6] in particular, substantially lower rates of alcohol related harms (deaths and hospitalisations) have been found amongst participants, compared to the general population.[7] Where it is possible to identify non-participants, findings of higher harm rates among the non-participants relative to the participants have been reported.[8, 9] A set of health studies conducted in Finland found that deaths due to alcohol related diseases, injuries and poisonings had the largest relative mortality differences between participants and non-participants for men, and were second largest for women, exceeded only by deaths due to suicides.[9] In Denmark, non-participants were found to have significantly increased hazard ratios for alcohol related hospitalisations and deaths relative to participants.[8] Under such circumstances, there is bias present in the participant sample and, as a consequence, in the derived estimates of alcohol consumption. Attempts to correct for such non-participation bias typically make use of weights based on socio-demographic characteristics,[10] however, this may not fully capture health differences. The success of the weighting is dependent on the extent to which those participating are representative of their subgroups of the population. For instance, individuals in harder to reach subgroups – such as younger men from disadvantaged backgrounds – that do participate, are unlikely to be representative of their entire demographic and so weighting does not resolve the bias.[11]

We have developed[11] and applied[6, 12] a dedicated methodology which uses additional health information from data linkage and reference to population data to adjust for non-participation bias. This methodology has previously been used to improve estimates of population-level alcohol consumption, although it could be applied to other health related behaviours of interest, such as tobacco smoking.

Briefly, the methodology uses information on the differences in sociodemographic characteristics and rates of (in the case of the previous application: alcohol-related) hospitalisations and deaths for the sample respondents and the general population to generate synthetic values for sociodemographic variables and rates of harm representing the ‘non-participants’. Multiple

imputation is then used to fill in values for the 'missing' variables collected in the health survey (alcohol consumption, in the case of the application) for these 'non-participants'. Multiple imputation has the flexibility to accommodate differences between participants and non-participants within groups defined by harm status as well as sociodemographic characteristics. Application of this methodology in Scotland found that mean alcohol consumption was between 14% and 53% higher for men after non-response bias was corrected for, depending on how extreme the differences in sex-specific mean weekly consumption between participants and non-participants were assumed to be, with little impact on estimates for women.[12]

This project aims to validate the methodology developed for addressing non-participation bias. Validation requires a setting whereby some true information on the individual non-participants of a health survey is known, and these can be compared to the synthetic observations generated by our methodology. Finland provides this opportunity as it maintains a nationally representative register which forms the sampling frame for surveys, and has the ability to inter-link sociodemographic information, morbidity and mortality databases, and survey responses at the individual level using personal identification code.[13] Therefore, through the use of this register, the sociodemographic, hospitalisation and death categories of the true non-participants are known (providing the "gold standard"). With the addition of the general population data, we are able to make indirect inference using the synthetic observations. We can then compare the results of the synthetic and true non-participants, allowing us to assess the validity of our existing methodology.

METHODS AND ANALYSIS

Health 2000 Survey data

The Health 2000 Survey (thl.fi/health2000) is a nationally representative health examination survey conducted in Finland between 2000 and 2001. A regional two-stage stratified cluster sampling strategy was used to identify approximately 8,000 persons aged 30 and over, in the main survey.[14] Figure 1 describes the Health 2000 sample, and the process of identifying the subsample for this analysis. The sample members aged 30 and over (n=8,028) were invited to participate in a home-based interview, to self-complete a health questionnaire, and to attend a health examination. The health questionnaire[15] comprised questions relating to living habits and environments, and included questions on the types, quantities and frequency of alcohol consumed over the past 12 months, from which we can derive an estimate of average weekly alcohol consumption, measured in grams per week. Of the persons aged 30 and over, 84.4% (n=6,736) completed the health questionnaire. These were considered to be participants for the purposes of this validation project, as the health questionnaire was the source of the outcome of interest – average weekly alcohol consumption. For

For peer review only

the purposes of our study, non-participants comprise those who had not completed the health questionnaire, as well as those who had not participated in any part of the survey, resulting in a total of 1,243 (15.6%) non-participants. Given that multiple comorbidities are more common at the older ages, and advanced ages are likely to have a lowering tolerance to alcohol and a change in drinking patterns,[16, 17] the subsample for this analysis, described in Figure 1, will be limited to those aged 30 to 79 years at the start of follow-up.

[Figure 1]

The selected Health 2000 sample were drawn from the Population Register Centre dataset, held by the Social Insurance Institution (Kela) of Finland.[18] The outcome of interest – average weekly alcohol consumption – is derived using the self-reported frequency and quantity of three types (beer, wine and spirits) of alcohol consumed in the past month/12 months, collected in Health Questionnaire 1.

Sampling weights were calculated by the Health 2000 study team, and were estimated based on a design weight, health centre district and university hospital district indicators, 10-year age group, gender, and native language (Finnish or Swedish). The design weights took the sampling design into account, including stratum and cluster specific inclusion probabilities, and the oversampling for the over 80's.[19] Sampling weights were estimated for persons who had participated in at least one stage of data collection; including home interviews, health exams, and questionnaires. Therefore weights were available for all participants and some non-participants, as defined in this analysis. The weights will be retained for the participants, and all non-participants will have their weight set to 1.

General population data

An 11% sample of the contemporaneous total Finnish population obtained from Statistics Finland, aggregated by age-group, sex and socioeconomic characteristics is available as the reference comparator for this analysis, linked to records of alcohol-related hospitalisations, and all cause deaths available from 20 October 2000 (median start of follow-up date for Health 2000 survey cohort). The population sample is restricted to those aged 30 to 79 years on that date. To negate the wait involved in applying for unnecessary individual level data, sociodemographic-specific age standardised rates of alcohol-related harms are being provided to us by Statistics Finland for cause-specific deaths, and alcohol-related hospitalisations by the National Institute for Health and Welfare.

Linked Health 2000-deaths/hospitalisations

In Finland, nationally representative administrative registers, as for other Nordic countries, enables the linkage of both participants and non-participants of the Health 2000 survey, and the 11% sample of the contemporaneous population to hospitalisation (alcohol related) and death (all causes and

For peer review only

alcohol related) records, as well as sociodemographic variables. The Classification of Socio-economic Groups 1989,[20] which classifies persons based on their occupation is available for the analysis, along with age, sex and region of residence. The Classification of Socio-economic Groups for both the Health 2000 sample and the 11% sample have been sourced from Statistics Finland, ensuring that they are measured at approximately the same time across the population. Linkage provides information on alcohol-related hospitalisations (date of event, ICD codes) and all-cause and alcohol-related deaths (date of death, ICD codes) from which an indicator of alcohol-related harm can be derived. Follow-up for hospitalisations and deaths of the Health 2000 sample is available until 31 December 2015, whereas follow-up is limited to the end of 2012 for the general population sample. Therefore, to ensure like-for-like comparisons are made, follow-up for all analyses will be truncated at 31 December 2012.

Inclusion criteria

To be included within this validation study, participants of the Health 2000 survey had to have been interviewed between the ages of 30 and 79 years. The lower age limit was constrained from the Health 2000 survey protocol, and the upper age limit was introduced as the overall Health 2000 survey design included an over-sample of those aged 80 and over at the time of selection.[14] The non-participants and contemporaneous population were selected for inclusion if they were aged 30 to 79 years at the start of follow-up (range August 2000 – November 2001 for Health 2000 cohort), or at 20 October 2000 (for contemporaneous population sample).

Statistical methodology

We aim to examine the differences in estimated average weekly alcohol consumption determined from the two methods: basing the alcohol consumption imputation on the actual sociodemographic and harm data at the individual level on the true non-participants versus basing the imputation on the synthetic observations on non-participants generated using the general population (Figure 2).

In doing this we will:

1. Quantify the differences in alcohol-related harm and all-cause mortality between survey participants and non-participants using rate ratios of alcohol-related harms using Poisson or negative binomial regression.
2.
    a. Perform multiple imputation to estimate values of average weekly alcohol consumption for non-participants, based on known age-group, sex, socioeconomic measures, deaths and hospitalisations due to alcohol.

For peer review only

b. Apply the existing methodology outlined in Gray et al. (2013)[11] and executed in Gorman et al. (2017)[12] to create the synthetic observations of sociodemographic measures, and rates of hospitalisation and deaths of non-participants based on a comparison of participants and the contemporaneous population and estimate non-participants' alcohol consumption using multiple imputation.

3. Examine how the estimates of alcohol consumption differ between the approaches taken in steps 2a and 2b.

[Figure 2]

Implications

This work has implications for the conduct and analysis of population-sampled surveys. Should the estimated average weekly alcohol consumptions from the two approaches be similar, the existing methodology can be applied to correct for bias arising from non-participation with greater confidence to a wide range of population health measures obtained through health surveys. Should the estimated consumptions differ between the two approaches, further investigations will be required, such as comparisons of the selected survey sample (participants and non-participants) to the population sample.

Practical/operational issues

The outcome of interest in this project, average alcohol consumed per week, is derived from self-reported drinking status (current, ex and never) and amounts of alcohol consumed by type (beer, wine and spirits). There are several instances where the responses provided conflict between questions, such as those who describe themselves as non-drinkers, but also report consuming alcohol within the last 12 months. Average weekly alcohol consumption was calculated by the Health 2000 project team, and this analysis follows the rules defined in their calculations. A sensitivity analysis will be performed exploring the effects of amending the drinking status and/or average amount consumed in conflicting cases.

The previous applications of this methodology[6, 12] were conducted in a setting in which it was natural to use an area level deprivation index as the socioeconomic measure. No official measure of area level deprivation exists for Finland, which leads us to using individual socioeconomic position for the validation exercise. Given that individual level measures of socioeconomic position are likely to be more informative than area based measures, and that relationships between consumption and alcohol-related harms have been found to be stronger using individual level measures,[21] the application of this methodology to settings with area based measures may require further validation.

For peer review only

Ethics and dissemination

The plans and protocol for the Health 2000 survey were reviewed by the National Public Health Institute's Ethical Committee in 1999, and approved by the Ethical Committee for Research in Epidemiology and Public Health at the Hospital District of Helsinki and Uusimaa (HUS) in 2000.

The members in the Health 2000 survey cohort were sent information letters prior to participating in the survey, which included a description of the study contents, rights of participant, and the possibility of later linkage to register data. Signed informed consent forms were required from all participants.[14] In Finland, survey data can be linked to registers if a) survey participants have provided informed consent for this, and b) register owner provides right for use of register data.[13] From survey non-participants no survey data exists, so linkage can be done with the permission from the register owner only.

In order to access Health 2000 files for secondary data analysis, as is being performed in this project, researchers were required to submit research plans for approval by the Health 2000 Scientific Advisory Board. Statistics Finland approved access to records of deaths and sociodemographic data, and the National Institute for Health and Welfare (THL) for hospitalisation data of this sample.

The outputs of the research will include two papers: the mortality differences between survey participants and non-participants (step 1), and a comparison of the inference from the two methods (steps 2 and 3).

Beneficiaries and target audiences

Research on alcohol consumption, and more broadly, methods to improve on estimates derived from health surveys, will be of interest to a range of both academic and non-academic audiences including users of survey data, epidemiologists, public health and policy researchers, and governmental organisations. The findings of this validation exercise will have implications for general survey conduct: particularly if the methodology is shown to be invalid, consideration could be given to basing sampling frames on sources which readily identify non-participants as well as participants and enable linkage to administrative records at the individual level. Should the results of the future analysis demonstrate the validity of the methodology, the approach will be of benefit in the evaluation and creation of public health policy in both local and international governments.

Data sharing statement

For peer review only

Access to the Health 2000 individual level survey responses can be requested via the Health 2000 homepage (thl.fi/health2000) from National Institute for Health and Welfare in Finland. Code used in the outputs will be made available in conjunction with their publication.

For peer review only

ADDITIONAL INFORMATION

Acknowledgements

We would like to thank the participants of the Health 2000 study, National Institute for Health and Welfare (THL) and Statistics Finland for the provision of the sociodemographic, hospitalisation and death data. Thanks in particular to Joonas J. J. Pitkänen from the Population Research Unit, Faculty of Social Sciences at the University of Helsinki for the preparation and provision of the population data.

Author Contributions

MAM prepared the first draft of this paper. The validation exercise was conceived by LG and AHL in discussion with PM. EG, TH and HR facilitated the acquisition of the survey and register-based data; PM facilitated the acquisition of the population sample data. HT contributed to the formulation of the approach. All authors contributed to all sections of the manuscript and approved the final version.

Funding

MAM, LG and AHL receive core funding from the Medical Research Council (MRC_MC_UU_12017/13) and the Scottish Government Chief Scientist Office (SPHSU13). TH, HR and HT are supported by the Academy of Finland under Grant 266251. PM is funded by the Academy of Finland.

Competing interests

MAM, PM, EG, HR, TH, HT, and LG have no conflicts to declare. AHL reports grants from Medical Research Council and Chief Scientist Office, during the conduct of the study.

Ethics approval

Research plans for this project have been approval by the Health 2000 Scientific Advisory Board. Access to register based sociodemographic variables and death data has been granted by Statistics Finland and to hospitalisation data by the National Institute for Health and Welfare (THL)

Figure Legends

Figure 1: Analytic sample selection process. Deaths and Hospitalisations to 31 December 2015.

Figure 2: Summary of proposed methodology

For peer review only

REFERENCES

1. Tolonen H, Helakorpi S, Talala K, et al. 25-year trends and socio-demographic differences in response rates: Finnish adult health behaviour survey. *Eur J Epidemiol* 2006;21(6):409-15.
2. Galea S, Tracy M. Participation Rates in Epidemiologic Studies. *Ann Epidemiol* 2007;17:643-53.
3. Mindell JS, Giampaoli S, Goesswald A, et al. Sample selection, recruitment and participation rates in health examination surveys in Europe – experience from seven national surveys. *BMC Med Res Methodol* 2015;15(1):78. doi: 10.1186/s12874-015-0072-4
4. Hara M, Sasaki S, Sobue T, et al. Comparison of cause-specific mortality between respondents and nonrespondents in a population-based prospective study. *J Clin Epidemiol* 2002;55(2):150-56. doi: 10.1016/S0895-4356(01)00431-0
5. Goldberg M, Chastang JF, Leclerc A, et al. Socioeconomic, demographic, occupational, and health factors associated with participation in a long-term epidemiologic survey: a prospective study of the French GAZEL cohort and its target population. *Am J Epidemiol* 2001;154(4):373-84. [published Online First: 2001/08/10]
6. Keyes KM, Rutherford C, Popham F, et al. How healthy are survey respondents compared with the general population? Using survey-linked death records to compare mortality outcomes. *Epidemiology* 2018;29(2):299-307. doi: 10.1097/ede.0000000000000775
7. Gorman E, Leyland AH, McCartney G, et al. Assessing the Representativeness of Population-Sampled Health Surveys Through Linkage to Administrative Data on Alcohol-Related Outcomes. *Am J Epidemiol* 2014;180(9):941-48. doi: 10.1093/aje/kwu207
8. Christensen AI, Ekholm O, Gray L, et al. What is wrong with non-respondents? Alcohol-, drug- and smoking-related mortality and morbidity in a 12-year follow-up study of respondents and non-respondents in the Danish Health and Morbidity Survey. *Addiction* 2015;110(9):1505-12. doi: 10.1111/add.12939
9. Jousilahti P, Salomaa V, Kuulasmaa K, et al. Total and cause specific mortality among participants and non-participants of population based health surveys: a comprehensive follow up of 54 372 Finnish men and women. *J Epidemiol Community Health* 2005;59(4):310-15. doi: 10.1136/jech.2004.024349
10. Gray L. The importance of post hoc approaches for overcoming non-response and attrition bias in population-sampled studies. *Soc Psychiatry Psychiatr Epidemiol* 2016;51(1):155-57. doi: 10.1007/s00127-015-1153-8
11. Gray L, McCartney G, White IR, et al. Use of record-linkage to handle non-response and improve alcohol consumption estimates in health survey data: a study protocol. *BMJ Open* 2013;3(3) doi: 10.1136/bmjopen-2013-002647
12. Gorman E, Leyland AH, McCartney G, et al. Adjustment for survey non-representativeness using record-linkage: refined estimates of alcohol consumption by deprivation in Scotland. *Addiction* 2017;112(7):1270-80. doi: 10.1111/add.13797
13. Gissler M, Haukka J. Finnish health and social welfare registers in epidemiological research. *Norsk Epidemiologi* 2004;14(1):113-20.
14. *Methodology Report: Health 2000 Survey*. Helsinki: KTL-National Public Health Institute, Finland Department of Health and Functional Capacity, 2008.
15. Health 2000. A Survey on Health and Functional Capacity in Finland: QUESTIONNAIRE 1, 2000. https://thl.fi/documents/189940/4108213/T2002_eng.pdf/83a57f85-0acb-4b25-af7d-29d7bf4b2282 (accessed 08/08/2018).
16. Hajat S, Haines A, Bulpitt C, et al. Patterns and determinants of alcohol consumption in people aged 75 years and older: results from the MRC trial of assessment and management of older people in the community. *Age Ageing* 2004;33(2):170-77. doi: 10.1093/ageing/afh046
17. Immonen S, Valvanne J, Pitkala KH. Prevalence of at-risk drinking among older adults and associated sociodemographic and health-related factors. *J Nutr Health Aging* 2011;15(9):789-94. [published Online First: 2011/11/18]

For peer review only

18. Niemelä H, Salminen K. Social security in Finland: Kela, Eläketurvakeskus, Työeläkevakuuttajat Tela ja sosiaali-ja terveysministeriö 2006.
19. Djerf K, Laiho J, Lehtonen R, et al. Weighting and Statistical Analysis. In: Heistaro S, ed. Methodology Report: Health 2000 Survey. Helsinki: KTL-National Public Health Institute, Finland Department of Health and Functional Capacity, 2008. http://urn.fi/URN:NBN:fi-fe201204193320.
20. Statistics Finland. Classification of Socio-economic Groups 1989: Statistics Finland; [Available from: https://www.stat.fi/meta/luokitukset/sosioekon_asema/001-1989/index_en.html accessed 17/05/2018.
21. Katikireddi SV, Whitley E, Lewsey J, et al. Socioeconomic status as an effect modifier of alcohol consumption and harm: analysis of linked cohort data. *The Lancet Public Health* 2017;2(6):e267-e76. doi: https://doi.org/10.1016/S2468-2667(17)30078-6

For peer review only

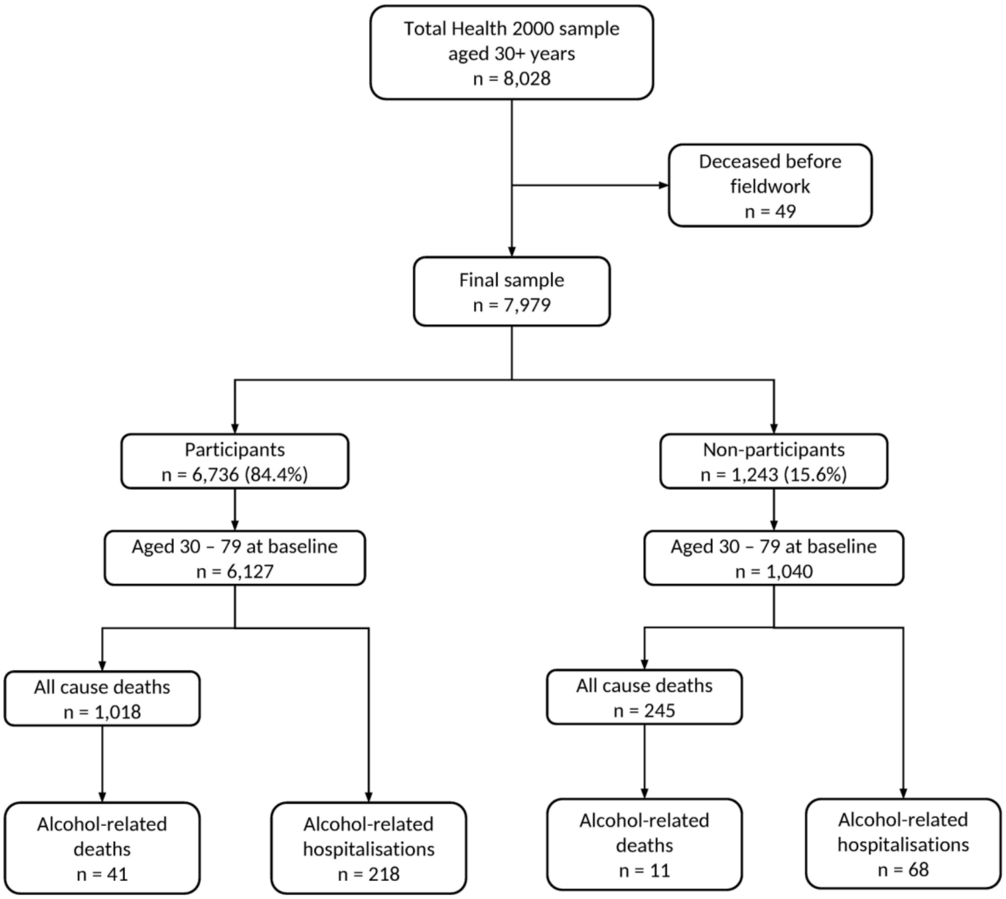

Analytic sample selection process. Deaths and Hospitalisations to 31 December 2015

150x134mm (300 x 300 DPI)

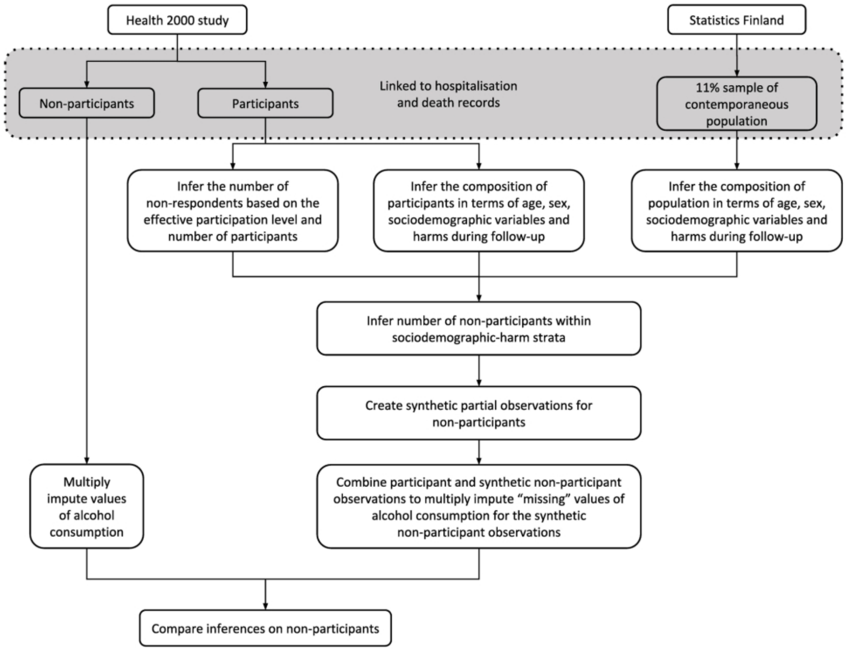

Summary of proposed methodology

190x142mm (300 x 300 DPI)

BMJ Open

Validation of non-participation bias methodology based on record-linked Finnish register-based health survey data: a protocol paper

Journal:	*BMJ Open*
Manuscript ID	bmjopen-2018-026187.R1
Article Type:	Protocol
Date Submitted by the Author:	09-Jan-2019
Complete List of Authors:	McMinn, Megan; University of Glasgow, MRC/CSO Social and Public Health Sciences Unit<br>Martikainen, Pekka; University of Helsinki, Faculty of Social Science, Population Research Unit<br>Gorman, Emma; Lancaster University, Department of Economics<br>Rissanen, Harri; National Institute for Health and Welfare, Department of Public Health Solutions<br>Härkänen, Tommi; National Institute for Health and Welfare, Department of Public Health Solutions<br>Tolonen, Hanna; National Institute for Health and Welfare , Department of Public Health Solutions<br>Leyland, Alastair; University of Glasgow, MRC/CSO Social and Public Health Sciences Unit<br>Gray, Linsay; University of Glasgow, MRC/CSO Social and Public Health Sciences Unit
**Primary Subject Heading**:	Research methods
Secondary Subject Heading:	Health policy, Public health
Keywords:	Protocols & guidelines < HEALTH SERVICES ADMINISTRATION & MANAGEMENT, PUBLIC HEALTH, STATISTICS & RESEARCH METHODS

SCHOLARONE™
Manuscripts

Validation of non-participation bias methodology based on record-linked Finnish register-based health survey data: a protocol paper

Megan A. McMinn, MRC/CSO Social and Public Health Sciences Unit, University of Glasgow, 200 Renfield Street, Glasgow, UK

Pekka Martikainen, Population Research Unit, Faculty of Social Science, University of Helsinki, Finland

Emma Gorman, Department of Economics, Lancaster University, Bailrigg, Lancaster, UK

Harri Rissanen, Public Health Solutions, National Institute for Health and Welfare (THL), Mannerheimintie 166, Helsinki, Finland

Tommi Härkänen, Public Health Solutions, National Institute for Health and Welfare (THL), Mannerheimintie 166, Helsinki, Finland

Hanna Tolonen, Public Health Solutions, National Institute for Health and Welfare (THL), Mannerheimintie 166, Helsinki, Finland

Alastair H. Leyland, MRC/CSO Social and Public Health Sciences Unit, University of Glasgow, 200 Renfield Street, Glasgow, UK

Linsay Gray, MRC/CSO Social and Public Health Sciences Unit, University of Glasgow, 200 Renfield Street, Glasgow, UK

Corresponding author: Megan McMinn

MRC/CSO Social and Public Health Sciences Unit

200 Renfield Street

Glasgow, G2 3AX

megan.mcminn@glasgow.ac.uk

Tel: 0141 535 7643

Word count: 2,531

For peer review only

ABSTRACT

Introduction

Decreasing participation levels in health surveys pose a threat to the validity of estimates intended to be representative of their target population. If participants and non-participants differ systematically, the results may be biased. The application of traditional non-response adjustment methods, such as weighting, can fail to correct for such biases, as estimates are typically based on the sociodemographic information available. Therefore, a dedicated methodology to infer on non-participants offers advancement by employing survey data linked to administrative health records, with reference to data on the general population. We aim to validate such a methodology in a register-based setting, where individual-level data on participants and non-participants are available, taking alcohol consumption estimation as the exemplar focus.

Methods and analysis

We make use of the selected sample of the Health 2000 survey conducted in Finland, and a separate register-based sample of the contemporaneous population, with follow-up until 2012. Finland has nationally representative administrative and health registers available for individual level record-linkage to the Health 2000 survey participants and invited non-participants, and the population sample. By comparing the population sample and the participants, synthetic observations representing the non-participants may be generated, as per the developed methodology. We can compare the distribution of the synthetic non-participants with the true distribution from the register data. Multiple imputation is then used to estimate alcohol consumption based on both the actual and synthetic data for non-participants, and the estimates can be compared to evaluate the methodology's performance.

Ethics and dissemination

Ethical approval and access to the Health 2000 survey data, and data from administrative and health registers has been given by the Health 2000 Scientific Advisory Board, Statistics Finland and the National Institute for Health and Welfare. The outputs will include two publications in public health and statistical methodology journals, and conference presentations.

For peer review only

ARTICLE SUMMARY

Strengths and Limitations of this study

This study will validate a dedicated methodology which aims to adjust for non-participation bias in health surveys through the use of record linkage.

We use an individual level dataset on the entire selected sample for a Finnish national health survey, from which the characteristics of non-participants can be identified, with linkage to morbidity and mortality records, providing the “gold standard” for the methodology validation process.

Previous applications of this methodology have been able to use data on the total population for comparison, this study is limited to a population sample available for this analysis.

The estimated gradient in the risk of alcohol-related harms may be stronger using individual measures of socioeconomic position than area level measures of deprivation; therefore these reference comparisons may not mirror the methodology based on less informative area-based measures.

This validation exercise is confined to assessing the reliability of inferring on non-participants from comparisons of the participants and the reference population; other aspects of the methodology such as the extent to which alcohol-related hospitalisations and deaths provide sufficient information to impute unknown alcohol consumption estimates are beyond the scope of this study.

For peer review only

INTRODUCTION

Health surveys enable the production of estimates of various health-related behaviours, such as smoking prevalence, levels of physical activity and alcohol consumption for entire populations, not confined to the sub-population in contact with health services. However the decreasing levels of participation in these surveys threaten their ability to provide reliable estimates.[1-3] The proportions of non-participation are typically not uniform across sociodemographic groups, meaning that selected groups, such as men or those from deprived backgrounds, are often underrepresented in health surveys.[4] Non-participation has also been found to correlate with higher rates of morbidity and mortality;[5, 6] in particular, substantially lower rates of alcohol related harms (deaths and hospitalisations) have been found amongst participants, compared to the general population.[7] Where it is possible to identify non-participants, findings of higher harm rates among the non-participants relative to the participants have been reported.[8, 9] A set of health studies conducted in Finland found that deaths due to alcohol related diseases, injuries and poisonings had the largest relative mortality differences between participants and non-participants for men, and were second largest for women, exceeded only by deaths due to suicides.[9] In Denmark, non-participants were found to have significantly increased hazard ratios for alcohol related hospitalisations and deaths relative to participants.[8] Under such circumstances, there is bias present in the participant sample and, as a consequence, in the derived estimates of alcohol consumption. Attempts to correct for such non-participation bias typically make use of weights based on socio-demographic characteristics,[10] however, this may not fully capture health differences. The success of the weighting is dependent on the extent to which those participating are representative of their subgroups of the population. For instance, individuals in harder to reach subgroups – such as younger men from disadvantaged backgrounds – that do participate, are unlikely to be representative of their entire demographic and so weighting does not resolve the bias.[11]

We have developed[11] and applied[6, 12] a dedicated methodology which uses additional health information from data linkage and reference to population data to adjust for non-participation bias. This methodology has previously been used to improve estimates of population-level alcohol consumption, although it could be applied to other health related behaviours of interest, such as tobacco smoking.

Briefly, the methodology makes inference on the non-participants by comparing the sociodemographic characteristics and rates of (in the case of the previous application: alcohol-related) hospitalisations and deaths in the survey participants, to the population, identifying any deviations in representativeness. Any differences point to non-participation in the respective

For peer review only - http://bmjopen.bmj.com/site/about/guidelines.xhtml

sociodemographic-health grouping. The number of synthetic observations on non-participants to generate in each sociodemographic-health group is based on the number of participants and the overall participation rate, with uncertainty due to sampling variation introduced through the use of repeated bootstrap samples and random rounding. Multiple imputation is then used to fill in values for the ‘missing’ variables collected in the health survey (alcohol consumption, in the case of the application) for these ‘non-participants’. Multiple imputation has the flexibility to accommodate differences between participants and non-participants within groups defined by harm status as well as sociodemographic characteristics. Application of this methodology in Scotland found that mean weekly alcohol consumption was between 14% and 53% higher for men after non-response bias was corrected for, depending on how extreme the differences in sex-specific mean weekly consumption between participants and non-participants were assumed to be, with little impact on estimates for women.[12]

This project aims to validate the methodology developed for addressing non-participation bias. Validation requires a setting whereby some true information on the individual non-participants of a health survey is known, and these can be compared to the synthetic observations generated by our methodology. Finland provides this opportunity as it maintains a nationally representative register which forms the sampling frame for surveys, and has the ability to inter-link sociodemographic information, morbidity and mortality databases, and survey responses at the individual level using personal identification codes.[13] Therefore, through the use of this register, the sociodemographic, hospitalisation and death categories of the true non-participants are known (providing the “gold standard”). With the addition of the general population data, we are able to make indirect inference using the synthetic observations. We can then compare the results of the synthetic and true non-participants, allowing us to assess the validity of our existing methodology.

METHODS AND ANALYSIS

Health 2000 Survey data

The Health 2000 Survey (thl.fi/health2000) is a nationally representative health examination survey conducted in Finland between 2000 and 2001. A regional two-stage stratified cluster sampling strategy was used to identify approximately 8,000 persons aged 30 and over, in the main survey.[14] Figure 1 describes the Health 2000 sample, and the process of identifying the subsample for this analysis. The sample members aged 30 and over (n=8,028) were invited to participate in a home-based interview, to self-complete a health questionnaire, and to attend a health examination. The health questionnaire[15] comprised questions relating to living habits and environments, and included questions on the types, quantities and frequency of alcohol consumed over the past 12 months,

For peer review only

from which we can derive an estimate of average weekly alcohol consumption, measured in grams per week. Of the persons aged 30 and over, 84.4% (n=6,736) completed the health questionnaire. These were considered to be participants for the purposes of this validation project, as the health questionnaire was the source of the outcome of interest – average weekly alcohol consumption. For the purposes of our study, non-participants comprise those who had not completed the health questionnaire, as well as those who had not participated in any part of the survey, resulting in a total of 1,243 (15.6%) non-participants. Given that multiple comorbidities are more common at the older ages, and advanced ages are likely to have a lowering tolerance to alcohol and a change in drinking patterns,[16, 17] the subsample for this analysis, described in Figure 1, will be limited to those aged 30 to 79 years at the start of follow-up.

[Figure 1]

The selected Health 2000 sample were drawn from the Population Register Centre dataset, held by the Social Insurance Institution (Kela) of Finland.[18] The outcome of interest – average weekly alcohol consumption – is derived using the self-reported frequency and quantity of three types (beer, wine and spirits) of alcohol consumed in the past month/12 months, collected in Health Questionnaire 1.

Sampling weights were calculated by the Health 2000 study team, and were estimated based on a design weight, health centre district and university hospital district indicators, 10-year age group, gender, and native language (Finnish or Swedish). The design weights took the sampling design into account, including stratum and cluster specific inclusion probabilities, and the oversampling for the over 80's.[19] Sampling weights were estimated for persons who had participated in at least one stage of data collection; including home interviews, health exams, and questionnaires. Therefore weights were available for some non-participants, as defined in this analysis, as they had participated in the home interview, but not the questionnaire collecting alcohol consumption, in addition to all participants. The weights will be retained for the participants, and all non-participants will have their weight set to the default value of 1.

General population data

An 11% sample of the contemporaneous total Finnish population aged 15 years and older, permanently living in Finland at the end of any of the years in 1987 to 2007 was constructed by Statistics Finland, and is available as the reference comparator for this analysis. This sample is supplemented with an additional 80% oversample of deaths occurring in 1988 to 2007. In line with the age limits used for the Health 2000 sample, the population sample is restricted to those aged 30 to 79 years alive on 20 October 2000 (median baseline date for Health 2000 survey cohort). Records

For peer review only

of all alcohol-related hospitalisations, and all-cause deaths occurring from 20 October 2000 to the end of 2012 were individually linked. To negate the wait involved in applying for unnecessary individual level data, sociodemographic-specific counts of alcohol-related harms and all cause deaths are being provided to us by Statistics Finland and the National Institute for Health and Welfare. These counts are weighted to account for the different sampling probabilities and the oversample of deaths.

Linked Health 2000-deaths/hospitalisations and educational attainment

In Finland, nationally representative administrative registers, as for other Nordic countries, enables the linkage of both participants and non-participants of the Health 2000 survey, and the 11% sample of the contemporaneous population to hospitalisation (alcohol related) and death (all causes and alcohol related) records, as well as sociodemographic variables. Educational attainment, dichotomised into four groups (basic, secondary, tertiary and post-graduate), is available for the analysis, along with age, sex and region of residence. Educational attainment for both the Health 2000 sample and the population sample have been sourced from Statistics Finland, ensuring that they are measured at approximately the same time across the population. Linkage provides information on alcohol-related in-patient hospitalisations (date of event, ICD codes) and all-cause and alcohol-related deaths (date of death, ICD codes) from which an indicator of alcohol-related harm can be derived. Follow-up for hospitalisations and deaths of the Health 2000 sample is available until 31 December 2015, whereas follow-up is limited to the end of 2012 for the general population sample. Therefore, to ensure like-for-like comparisons are made, follow-up for all analyses will be truncated at 31 December 2012.

Statistical methodology

We aim to examine the differences in estimated average weekly alcohol consumption determined from the two methods: basing the alcohol consumption imputation on the actual sociodemographic and harm data at the individual level on the true non-participants versus basing the imputation on the synthetic observations on non-participants generated using the general population (Figure 2).

In doing this we will:

1. Quantify the differences in alcohol-related harm and all-cause mortality between survey participants and non-participants, and survey participants and the population sample using rate ratios of alcohol-related harms using Poisson or Negative Binomial regression.
2.

For peer review only

a. Perform multiple imputation to estimate values of average weekly alcohol consumption for true non-participants, based on known age-group, sex, educational attainment, deaths and hospitalisations due to alcohol. This provides the 'gold standard'.

b. Apply the existing methodology outlined in Gray et al. (2013)[11] and executed in Gorman et al. (2017)[12] to create the synthetic observations of sociodemographic measures, and rates of hospitalisation and deaths of non-participants based on a comparison of participants and the contemporaneous population and estimate non-participants' alcohol consumption using multiple imputation.

3. Examine how the estimates of alcohol consumption differ between the approaches taken in steps 2a and 2b. This will be measured using the relative difference in mean weekly alcohol estimates, using the gold standard as the reference. Differences will be assessed overall, by sex, and by educational attainment. Repeated bootstrap samples will be used to generate a 95% confidence interval surrounding each relative difference, in order to assess how similar the two approaches are.

[Figure 2]

Implications

This work has implications for the conduct and analysis of population-sampled surveys. Should the estimated average weekly alcohol consumptions from the two approaches be similar, that is, the 95% confidence interval for the relative difference contains 0, we would consider the methodology a useful tool for correcting bias. The existing methodology could then be applied to correct for bias arising from non-participation with greater confidence to a wide range of population health measures obtained through health surveys, such as tobacco smoking or physical activity. Should the estimated consumptions differ between the two approaches, further investigations will be required, such as comparisons of the selected survey sample (participants and non-participants) to the population sample.

Practical/operational issues

The outcome of interest in this project, average alcohol consumed per week, is derived from self-reported drinking status (current, ex and never) and amounts of alcohol consumed by type (beer, wine and spirits). There are several instances where the responses provided conflict between questions, such as those who describe themselves as non-drinkers, but also report consuming alcohol within the last 12 months. Average weekly alcohol consumption was calculated by the

For peer review only

Health 2000 project team, and this analysis follows the rules defined in their calculations. A sensitivity analysis will be performed exploring the effects of amending the drinking status and/or average amount consumed in conflicting cases.

The previous applications of this methodology[6, 12] were conducted in a setting in which it was natural to use an area level deprivation index as the socioeconomic measure. No official measure of area level deprivation exists for Finland, which leads us to using educational attainment for the validation exercise. Given that individual level measures of socioeconomic position are likely to be more informative than area based measures, and that relationships between consumption and alcohol-related harms have been found to be stronger using individual level measures,[20] the application of this methodology to settings with area based measures may require further validation.

Ethics and dissemination

The plans and protocol for the Health 2000 survey were reviewed by the National Public Health Institute's Ethical Committee in 1999, and approved by the Ethical Committee for Research in Epidemiology and Public Health at the Hospital District of Helsinki and Uusimaa (HUS) in 2000.

The members in the Health 2000 survey cohort were sent information letters prior to participating in the survey, which included a description of the study contents, rights of participant, and the possibility of later linkage to register data. Signed informed consent forms were required from all participants.[14] In Finland, survey data can be linked to registers if a) survey participants have provided informed consent for this, and b) register owner provides right for use of register data.[13] From survey non-participants no survey data exists, so linkage can be done with the permission from the register owner only.

In order to access Health 2000 files for secondary data analysis, as is being performed in this project, researchers were required to submit research plans for approval by the Health 2000 Scientific Advisory Board. Statistics Finland approved access to records of deaths and sociodemographic data, and the National Institute for Health and Welfare (THL) for hospitalisation data of this sample.

The outputs of the research will include two papers: the mortality differences between survey participants and non-participants (step 1), and a comparison of the inference from the two methods (steps 2 and 3).

Beneficiaries and target audiences

Research on alcohol consumption, and more broadly, methods to improve on estimates derived from health surveys, will be of interest to a range of both academic and non-academic audiences

For peer review only

including users of survey data, epidemiologists, public health and policy researchers, and governmental organisations. The findings of this validation exercise will have implications for general survey conduct: particularly if the methodology is shown to be invalid, consideration could be given to basing sampling frames on sources which readily identify non-participants as well as participants and enable linkage to administrative records at the individual level. Should the results of the future analysis demonstrate the validity of the methodology, the approach will be of benefit in the evaluation and creation of public health policy in both local and international governments.

Patient and Public Involvement

In this research data from general population, not on patients, was used. This analysis utilised two large pseudonymised record-linked administrative datasets with no possibility of direct participant contact beyond their initial participation in the Health 2000 study, due to data protection restrictions. Participants were not invited to contribute to the writing or editing of this document for readability or accuracy.

Data sharing statement

Access to the Health 2000 individual level survey responses can be requested via the Health 2000 homepage (thl.fi/health2000) from National Institute for Health and Welfare in Finland. Code used in the outputs will be made available in conjunction with their publication.

For peer review only

ADDITIONAL INFORMATION

Acknowledgements

We would like to thank the participants of the Health 2000 study, National Institute for Health and Welfare (THL) and Statistics Finland for the provision of the sociodemographic, hospitalisation and death data. Thanks in particular to Joonas J. J. Pitkänen from the Population Research Unit, Faculty of Social Sciences at the University of Helsinki for the preparation and provision of the population data.

Author Contributions

MAM prepared the first draft of this paper. The validation exercise was conceived by LG and AHL in discussion with PM. EG, TH and HR facilitated the acquisition of the survey and register-based data; PM facilitated the acquisition of the population sample data. HT contributed to the formulation of the approach. All authors contributed to all sections of the manuscript and approved the final version.

Funding

MAM, LG and AHL receive core funding from the Medical Research Council (MRC_MC_UU_12017/13) and the Scottish Government Chief Scientist Office (SPHSU13). TH, HR and HT are supported by the Academy of Finland under Grant 266251. PM is funded by the Academy of Finland.

Competing interests

MAM, PM, EG, HR, TH, HT, and LG have no conflicts to declare. AHL reports grants from Medical Research Council and Chief Scientist Office, during the conduct of the study.

Ethics approval

Research plans for this project have been approval by the Health 2000 Scientific Advisory Board. Access to register based sociodemographic variables and death data has been granted by Statistics Finland and to hospitalisation data by the National Institute for Health and Welfare (THL)

Figure Legends

Figure 1: Analytic sample selection process. Deaths and Hospitalisations to 31 December 2015.

Figure 2: Summary of proposed methodology

For peer review only

REFERENCES

1. Tolonen H, Helakorpi S, Talala K, et al. 25-year trends and socio-demographic differences in response rates: Finnish adult health behaviour survey. *Eur J Epidemiol* 2006;21(6):409-15.
2. Galea S, Tracy M. Participation Rates in Epidemiologic Studies. *Ann Epidemiol* 2007;17:643-53.
3. Mindell JS, Giampaoli S, Goesswald A, et al. Sample selection, recruitment and participation rates in health examination surveys in Europe – experience from seven national surveys. *BMC Med Res Methodol* 2015;15(1):78. doi: 10.1186/s12874-015-0072-4
4. Hara M, Sasaki S, Sobue T, et al. Comparison of cause-specific mortality between respondents and nonrespondents in a population-based prospective study. *J Clin Epidemiol* 2002;55(2):150-56. doi: 10.1016/S0895-4356(01)00431-0
5. Goldberg M, Chastang JF, Leclerc A, et al. Socioeconomic, demographic, occupational, and health factors associated with participation in a long-term epidemiologic survey: a prospective study of the French GAZEL cohort and its target population. *Am J Epidemiol* 2001;154(4):373-84. [published Online First: 2001/08/10]
6. Keyes KM, Rutherford C, Popham F, et al. How healthy are survey respondents compared with the general population? Using survey-linked death records to compare mortality outcomes. *Epidemiology* 2018;29(2):299-307. doi: 10.1097/ede.0000000000000775
7. Gorman E, Leyland AH, McCartney G, et al. Assessing the Representativeness of Population-Sampled Health Surveys Through Linkage to Administrative Data on Alcohol-Related Outcomes. *Am J Epidemiol* 2014;180(9):941-48. doi: 10.1093/aje/kwu207
8. Christensen AI, Ekholm O, Gray L, et al. What is wrong with non-respondents? Alcohol-, drug- and smoking-related mortality and morbidity in a 12-year follow-up study of respondents and non-respondents in the Danish Health and Morbidity Survey. *Addiction* 2015;110(9):1505-12. doi: 10.1111/add.12939
9. Jousilahti P, Salomaa V, Kuulasmaa K, et al. Total and cause specific mortality among participants and non-participants of population based health surveys: a comprehensive follow up of 54 372 Finnish men and women. *J Epidemiol Community Health* 2005;59(4):310-15. doi: 10.1136/jech.2004.024349
10. Gray L. The importance of post hoc approaches for overcoming non-response and attrition bias in population-sampled studies. *Soc Psychiatry Psychiatr Epidemiol* 2016;51(1):155-57. doi: 10.1007/s00127-015-1153-8
11. Gray L, McCartney G, White IR, et al. Use of record-linkage to handle non-response and improve alcohol consumption estimates in health survey data: a study protocol. *BMJ Open* 2013;3(3) doi: 10.1136/bmjopen-2013-002647
12. Gorman E, Leyland AH, McCartney G, et al. Adjustment for survey non-representativeness using record-linkage: refined estimates of alcohol consumption by deprivation in Scotland. *Addiction* 2017;112(7):1270-80. doi: 10.1111/add.13797
13. Gissler M, Haukka J. Finnish health and social welfare registers in epidemiological research. *Norsk Epidemiologi* 2004;14(1):113-20.
14. *Methodology Report: Health 2000 Survey*. Helsinki: KTL-National Public Health Institute, Finland Department of Health and Functional Capacity, 2008.
15. Health 2000. A Survey on Health and Functional Capacity in Finland: QUESTIONNAIRE 1, 2000. https://thl.fi/documents/189940/4108213/T2002_eng.pdf/83a57f85-0acb-4b25-af7d-29d7bf4b2282 (accessed 08/08/2018).
16. Hajat S, Haines A, Bulpitt C, et al. Patterns and determinants of alcohol consumption in people aged 75 years and older: results from the MRC trial of assessment and management of older people in the community. *Age Ageing* 2004;33(2):170-77. doi: 10.1093/ageing/afh046
17. Immonen S, Valvanne J, Pitkala KH. Prevalence of at-risk drinking among older adults and associated sociodemographic and health-related factors. *J Nutr Health Aging* 2011;15(9):789-94. [published Online First: 2011/11/18]

For peer review only

18. Niemelä H, Salminen K. Social security in Finland: Kela, Eläketurvakeskus, Työeläkevakuuttajat Tela ja sosiaali-ja terveysministeriö 2006.
19. Djerf K, Laiho J, Lehtonen R, et al. Weighting and Statistical Analysis. In: Heistaro S, ed. Methodology Report: Health 2000 Survey. Helsinki: KTL-National Public Health Institute, Finland Department of Health and Functional Capacity, 2008. http://urn.fi/URN:NBN:fi-fe201204193320.
20. Katikireddi SV, Whitley E, Lewsey J, et al. Socioeconomic status as an effect modifier of alcohol consumption and harm: analysis of linked cohort data. *The Lancet Public Health* 2017;2(6):e267-e76. doi: https://doi.org/10.1016/S2468-2667(17)30078-6

For peer review only

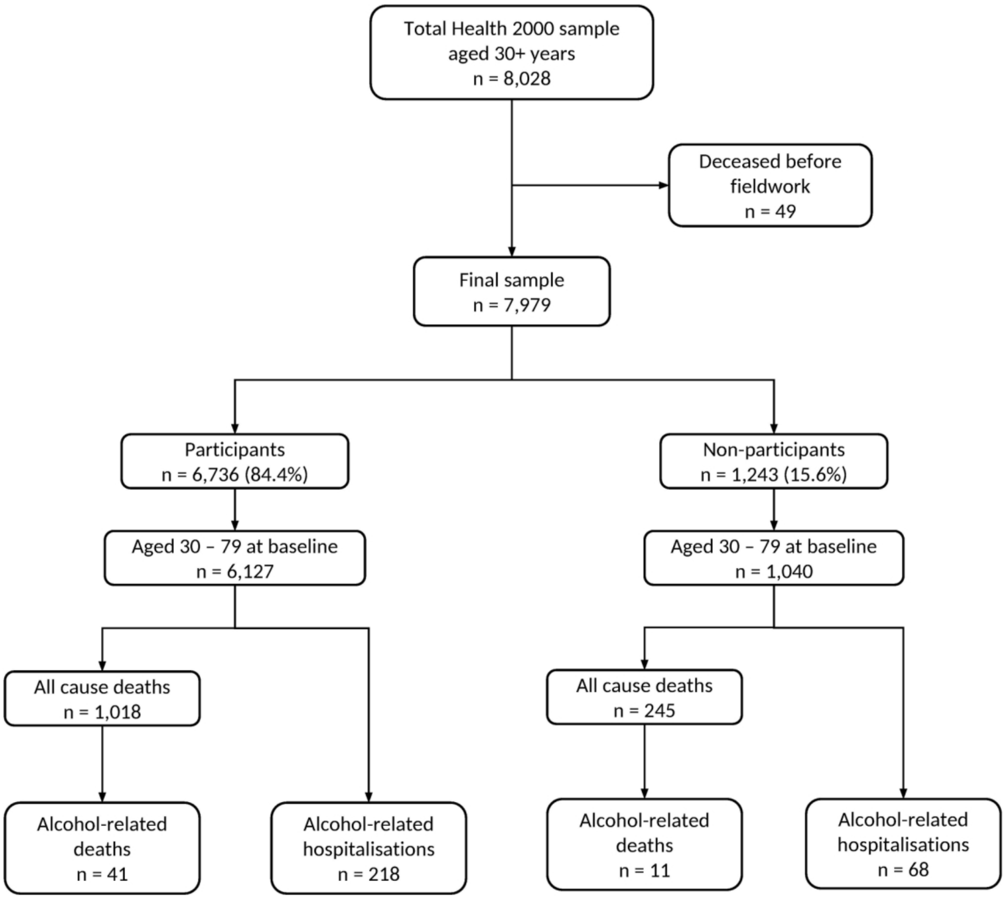

Analytic sample selection process. Deaths and Hospitalisations to 31 December 2015

150x134mm (300 x 300 DPI)

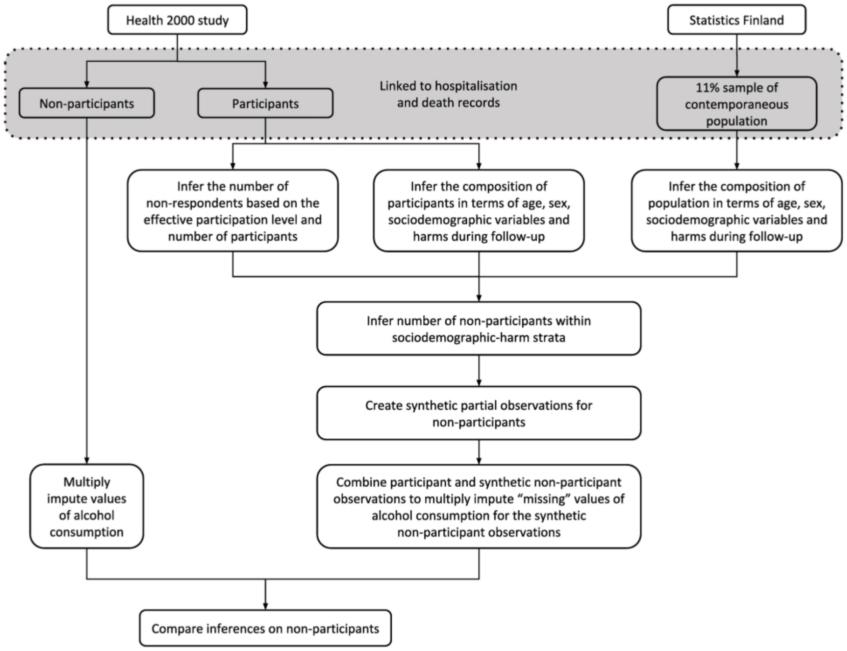

Summary of proposed methodology

190x142mm (300 x 300 DPI)

Validation of non-participation bias methodology based on record-linked Finnish register-based health survey data: a protocol paper

Journal:	*BMJ Open*
Manuscript ID	bmjopen-2018-026187.R2
Article Type:	Protocol
Date Submitted by the Author:	04-Mar-2019
Complete List of Authors:	McMinn, Megan; University of Glasgow, MRC/CSO Social and Public Health Sciences Unit<br>Martikainen, Pekka; University of Helsinki, Population Research Unit, Faculty of Social Science<br>Gorman, Emma; Lancaster University, Department of Economics<br>Rissanen, Harri; National Institute for Health and Welfare, Public Health Solutions<br>Härkänen, Tommi; National Institute for Health and Welfare, Public Health Solutions<br>Tolonen, Hanna; National Institute for Health and Welfare, Public Health Solutions<br>Leyland, Alastair; University of Glasgow, MRC/CSO Social and Public Health Sciences Unit<br>Gray, Linsay; University of Glasgow, MRC/CSO Social and Public Health Sciences Unit
**Primary Subject Heading**:	Research methods
Secondary Subject Heading:	Health policy, Public health
Keywords:	Protocols & guidelines < HEALTH SERVICES ADMINISTRATION & MANAGEMENT, PUBLIC HEALTH, STATISTICS & RESEARCH METHODS

SCHOLARONE™
Manuscripts

Validation of non-participation bias methodology based on record-linked Finnish register-based health survey data: a protocol paper

Megan A. McMinn, MRC/CSO Social and Public Health Sciences Unit, University of Glasgow, 200 Renfield Street, Glasgow, UK

Pekka Martikainen, Population Research Unit, Faculty of Social Science, University of Helsinki, Finland

Emma Gorman, Department of Economics, Lancaster University, Bailrigg, Lancaster, UK

Harri Rissanen, Public Health Solutions, National Institute for Health and Welfare (THL), Mannerheimintie 166, Helsinki, Finland

Tommi Härkänen, Public Health Solutions, National Institute for Health and Welfare (THL), Mannerheimintie 166, Helsinki, Finland

Hanna Tolonen, Public Health Solutions, National Institute for Health and Welfare (THL), Mannerheimintie 166, Helsinki, Finland

Alastair H. Leyland, MRC/CSO Social and Public Health Sciences Unit, University of Glasgow, 200 Renfield Street, Glasgow, UK

Linsay Gray, MRC/CSO Social and Public Health Sciences Unit, University of Glasgow, 200 Renfield Street, Glasgow, UK

Corresponding author: Megan McMinn

MRC/CSO Social and Public Health Sciences Unit

200 Renfield Street

Glasgow, G2 3AX

megan.mcminn@glasgow.ac.uk

Tel: 0141 535 7643

Word count: 2,711

For peer review only

ABSTRACT

Introduction

Decreasing participation levels in health surveys pose a threat to the validity of estimates intended to be representative of their target population. If participants and non-participants differ systematically, the results may be biased. The application of traditional non-response adjustment methods, such as weighting, can fail to correct for such biases, as estimates are typically based on the sociodemographic information available. Therefore, a dedicated methodology to infer on non-participants offers advancement by employing survey data linked to administrative health records, with reference to data on the general population. We aim to validate such a methodology in a register-based setting, where individual-level data on participants and non-participants are available, taking alcohol consumption estimation as the exemplar focus.

Methods and analysis

We make use of the selected sample of the Health 2000 survey conducted in Finland, and a separate register-based sample of the contemporaneous population, with follow-up until 2012. Finland has nationally representative administrative and health registers available for individual level record-linkage to the Health 2000 survey participants and invited non-participants, and the population sample. By comparing the population sample and the participants, synthetic observations representing the non-participants may be generated, as per the developed methodology. We can compare the distribution of the synthetic non-participants with the true distribution from the register data. Multiple imputation is then used to estimate alcohol consumption based on both the actual and synthetic data for non-participants, and the estimates can be compared to evaluate the methodology's performance.

Ethics and dissemination

Ethical approval and access to the Health 2000 survey data, and data from administrative and health registers has been given by the Health 2000 Scientific Advisory Board, Statistics Finland and the National Institute for Health and Welfare. The outputs will include two publications in public health and statistical methodology journals, and conference presentations.

For peer review only

ARTICLE SUMMARY

Strengths and Limitations of this study

This study will validate a dedicated methodology which aims to adjust for non-participation bias in health surveys through the use of record linkage.

We use an individual level dataset on the entire selected sample for a Finnish national health survey, from which the characteristics of non-participants can be identified, with linkage to morbidity and mortality records, providing the “gold standard” for the methodology validation process.

Previous applications of this methodology have been able to use data on the total population for comparison, this study is limited to a population sample available for this analysis.

The estimated gradient in the risk of alcohol-related harms may be stronger using individual measures of socioeconomic position than area level measures of deprivation; therefore these reference comparisons may not mirror the methodology based on less informative area-based measures.

This validation exercise is confined to assessing the reliability of inferring on non-participants from comparisons of the participants and the reference population; other aspects of the methodology such as the extent to which alcohol-related hospitalisations and deaths provide sufficient information to impute unknown alcohol consumption estimates are beyond the scope of this study.

For peer review only

INTRODUCTION

Health surveys enable the production of estimates of various health-related behaviours, such as smoking prevalence, levels of physical activity and alcohol consumption for entire populations, not confined to the sub-population in contact with health services. However the decreasing levels of participation in these surveys threaten their ability to provide reliable estimates.[1-3] The proportions of non-participation are typically not uniform across sociodemographic groups, meaning that selected groups, such as men or those from deprived backgrounds, are often underrepresented in health surveys.[4] Non-participation has also been found to correlate with higher rates of morbidity and mortality;[5, 6] in particular, substantially lower rates of alcohol related harms (deaths and hospitalisations) have been found amongst participants, compared to the general population.[7] Where it is possible to identify non-participants, findings of higher harm rates among the non-participants relative to the participants have been reported.[8, 9] A set of health studies conducted in Finland found that deaths due to alcohol related diseases, injuries and poisonings had the largest relative mortality differences between participants and non-participants for men, and were second largest for women, exceeded only by deaths due to suicides.[9] In Denmark, non-participants were found to have significantly increased hazard ratios for alcohol related hospitalisations and deaths relative to participants.[8] Under such circumstances, there is bias present in the participant sample and, as a consequence, in the derived estimates of alcohol consumption. Attempts to correct for such non-participation bias typically make use of weights based on socio-demographic characteristics,[10] however, this may not fully capture health differences. The success of the weighting is dependent on the extent to which those participating are representative of their subgroups of the population. For instance, individuals in harder to reach subgroups – such as younger men from disadvantaged backgrounds – that do participate, are unlikely to be representative of their entire demographic and so weighting does not resolve the bias.[11]

We have developed[11] and applied[6, 12] a dedicated methodology which uses additional health information from data linkage and reference to population data to adjust for non-participation bias. This methodology has previously been used to improve estimates of population-level alcohol consumption, although it could be applied to other health related behaviours of interest, such as tobacco smoking.

Briefly, the methodology makes inference on the non-participants by comparing the sociodemographic characteristics and rates of (in the case of the previous application: alcohol-related) hospitalisations and deaths in the survey participants, to the population, identifying any deviations in representativeness. Any differences point to non-participation in the respective

For peer review only - http://bmjopen.bmj.com/site/about/guidelines.xhtml

sociodemographic-health grouping. The number of synthetic observations on non-participants to generate in each sociodemographic-health group is based on the number of participants and the overall participation rate, with uncertainty due to sampling variation introduced through the use of repeated bootstrap samples and random rounding. Multiple imputation is then used to fill in values for the 'missing' variables collected in the health survey (alcohol consumption, in the case of the application) for these 'non-participants'. Multiple imputation has the flexibility to accommodate differences between participants and non-participants within groups defined by harm status as well as sociodemographic characteristics. Application of this methodology in Scotland found that mean weekly alcohol consumption was between 14% and 53% higher for men after non-response bias was corrected for, depending on how extreme the differences in sex-specific mean weekly consumption between participants and non-participants were assumed to be, with little impact on estimates for women.[12]

This project aims to validate the methodology developed for addressing non-participation bias. More specifically, to evaluate whether it is valid to infer on the non-participants from comparisons of the participants and a total register based population sample without non-response. Validation requires a setting whereby some true information on the individual non-participants of a health survey is known, and these can be compared to the synthetic observations generated by our methodology. Finland provides this opportunity as it maintains a nationally representative register which forms the sampling frame for surveys, and has the ability to inter-link sociodemographic information, morbidity and mortality databases, and survey responses at the individual level using personal identification codes.[13] Therefore, through the use of this register, the sociodemographic, hospitalisation and death categories of the true non-participants are known (providing the "gold standard"). With the addition of the general population data, we are able to make indirect inference using the synthetic observations. We can then compare the results of the synthetic and true non-participants, allowing us to assess the validity of our existing methodology.

For peer review only

METHODS AND ANALYSIS

Health 2000 Survey data

The Health 2000 Survey (thl.fi/health2000) is a nationally representative health examination survey conducted in Finland between 2000 and 2001. A regional two-stage stratified cluster sampling strategy was used to identify approximately 8,000 persons aged 30 and over, in the main survey.[14] Figure 1 describes the Health 2000 sample, and the process of identifying the subsample for this analysis. The sample members aged 30 and over (n=8,028) were invited to participate in a home-based interview, to self-complete a health questionnaire, and to attend a health examination. The

health questionnaire[15] comprised questions relating to living habits and environments, and included questions on the types, quantities and frequency of alcohol consumed over the past 12 months, from which we can derive an estimate of average weekly alcohol consumption, measured in grams per week. Of the persons aged 30 and over, 84.4% (n=6,736) completed the health questionnaire. These were considered to be participants for the purposes of this validation project, as the health questionnaire was the source of the outcome of interest – average weekly alcohol consumption. For the purposes of our study, non-participants comprise those who had not completed the health questionnaire, as well as those who had not participated in any part of the survey, resulting in a total of 1,243 (15.6%) non-participants. Given that multiple comorbidities are more common at the older ages, and advanced ages are likely to have a lowering tolerance to alcohol and a change in drinking patterns,[16, 17] the subsample for this analysis, described in Figure 1, will be limited to those aged 30 to 79 years at the start of follow-up.

[Figure 1]

The selected Health 2000 sample were drawn from the Population Register Centre dataset, held by the Social Insurance Institution (Kela) of Finland.[18] The outcome of interest – average weekly alcohol consumption – is derived using the self-reported frequency and quantity of three types (beer, wine and spirits) of alcohol consumed in the past month/12 months, collected in Health Questionnaire 1.

Sampling weights were calculated by the Health 2000 study team, and were estimated based on a design weight, health centre district and university hospital district indicators, 10-year age group, gender, and native language (Finnish or Swedish). The design weights took the sampling design into account, including stratum and cluster specific inclusion probabilities, and the oversampling for the over 80's.[19] Sampling weights were estimated for persons who had participated in at least one stage of data collection; including home interviews, health exams, and questionnaires. Therefore weights were available for some non-participants, as defined in this analysis, as they had participated in the home interview, but not the questionnaire collecting alcohol consumption, in addition to all participants. The weights will be retained for the participants, and all non-participants will have their weight set to the default value of 1.

General population data

An 11% sample of the contemporaneous total Finnish population aged 15 years and older, permanently living in Finland at the end of any of the years in 1987 to 2007 was constructed by Statistics Finland, and is available as the reference comparator for this analysis. This sample is supplemented with an additional 80% oversample of deaths occurring in 1988 to 2007. In line with

For peer review only

the age limits used for the Health 2000 sample, the population sample is restricted to those aged 30 to 79 years alive on 20 October 2000 (median baseline date for Health 2000 survey cohort). Records of all alcohol-related hospitalisations, and all-cause deaths occurring from 20 October 2000 to the end of 2012 were individually linked. To negate the wait involved in applying for unnecessary individual level data, sociodemographic-specific counts of alcohol-related harms and all cause deaths are being provided to us by Statistics Finland and the National Institute for Health and Welfare. These counts are weighted to account for the different sampling probabilities and the oversample of deaths.

Linked Health 2000-deaths/hospitalisations and educational attainment

In Finland, nationally representative administrative registers, as for other Nordic countries, enables the linkage of both participants and non-participants of the Health 2000 survey, and the 11% sample of the contemporaneous population to hospitalisation (alcohol related) and death (all causes and alcohol related) records, as well as sociodemographic variables. Educational attainment, dichotomised into four groups (basic, secondary, tertiary and post-graduate), is available for the analysis, along with age, sex and region of residence. Educational attainment for both the Health 2000 sample and the population sample have been sourced from Statistics Finland, ensuring that they are measured at approximately the same time across the population. Linkage provides information on alcohol-related in-patient hospitalisations (date of event, ICD codes) and all-cause and alcohol-related deaths (date of death, ICD codes) from which an indicator of alcohol-related harm can be derived. Follow-up for hospitalisations and deaths of the Health 2000 sample is available until 31 December 2015, whereas follow-up is limited to the end of 2012 for the general population sample. Therefore, to ensure like-for-like comparisons are made, follow-up for all analyses will be truncated at 31 December 2012.

For peer review only

Statistical methodology

We aim to examine the differences in estimated average weekly alcohol consumption determined from the two methods: basing the alcohol consumption imputation on the actual sociodemographic and harm data at the individual level on the true non-participants versus basing the imputation on the synthetic observations on non-participants generated using the general population (Figure 2).

In doing this we will:

1. Quantify the differences in alcohol-related harm and all-cause mortality between survey participants and non-participants, and survey participants and the population sample using rate ratios of alcohol-related harms using Poisson or Negative Binomial regression.

2.

   a. Perform multiple imputation to estimate values of average weekly alcohol consumption for true non-participants. This imputation will use the known age-group, sex, educational attainment, deaths and hospitalisations due to alcohol for the participants and non-participants, obtained from administrative data on the total sampling frame, and the known alcohol consumption of the participants alone, obtained from the Health 2000 survey. This provides the 'gold standard'.
   b. Apply the existing methodology outlined in Gray et al. (2013)[11] and executed in Gorman et al. (2017)[12] to create the synthetic observations of sociodemographic measures, and rates of hospitalisation and deaths of non-participants based on a comparison of participants and a contemporaneous total register based population sample without non-response. Multiple imputation is used to estimate these inferred non-participants' alcohol consumption.

3. Examine how the estimates of alcohol consumption differ between the approaches taken in steps 2a and 2b. This will be measured using the relative difference in mean weekly alcohol estimates, using the gold standard as the reference. Differences will be assessed overall, by sex, and by educational attainment. Repeated bootstrap samples will be used to generate a 95% confidence interval surrounding each relative difference, in order to assess how similar the two approaches are. The proportions of true and inferred non-participants within each age-sex-education-harm-mortality group can similarly be compared to assess how successfully the simulated observations on non-participants reflect the true non-participants.

[Figure 2]

Implications

This work has implications for the conduct and analysis of population-sampled surveys. Should the estimated average weekly alcohol consumptions from the two approaches be similar, that is, if the relative difference is smaller than the minimum acceptability limit of 5%, we would consider the methodology a useful tool for correcting bias. The bootstrapped 95% confidence intervals provide guidance as to the statistical significance of the difference. The existing methodology could then be applied to correct for bias arising from non-participation with greater confidence to a wide range of population health measures obtained through health surveys, such as tobacco smoking or physical activity. Should the estimated consumptions differ between the two approaches, further

For peer review only

investigations will be required, such as comparisons of the selected survey sample (participants and non-participants) to the population sample.

Practical/operational issues

The outcome of interest in this project, average alcohol consumed per week, is derived from self-reported drinking status (current, ex and never) and amounts of alcohol consumed by type (beer, wine and spirits). There are several instances where the responses provided conflict between questions, such as those who describe themselves as non-drinkers, but also report consuming alcohol within the last 12 months. Average weekly alcohol consumption was calculated by the Health 2000 project team, and this analysis follows the rules defined in their calculations. A sensitivity analysis will be performed exploring the effects of amending the drinking status and/or average amount consumed in conflicting cases.

The previous applications of this methodology[6, 12] were conducted in a setting in which it was natural to use an area level deprivation index as the socioeconomic measure. No official measure of area level deprivation exists for Finland, which leads us to using educational attainment for the validation exercise. Given that individual level measures of socioeconomic position are likely to be more informative than area based measures, and that relationships between consumption and alcohol-related harms have been found to be stronger using individual level measures,[20] the application of this methodology to settings with area based measures may require further validation.

Ethics and dissemination

The plans and protocol for the Health 2000 survey were reviewed by the National Public Health Institute's Ethical Committee in 1999, and approved by the Ethical Committee for Research in Epidemiology and Public Health at the Hospital District of Helsinki and Uusimaa (HUS) in 2000.

The members in the Health 2000 survey cohort were sent information letters prior to participating in the survey, which included a description of the study contents, rights of participant, and the possibility of later linkage to register data. Signed informed consent forms were required from all participants.[14] In Finland, survey data can be linked to registers if a) survey participants have provided informed consent for this, and b) register owner provides right for use of register data.[13] From survey non-participants no survey data exists, so linkage can be done with the permission from the register owner only.

In order to access Health 2000 files for secondary data analysis, as is being performed in this project, researchers were required to submit research plans for approval by the Health 2000 Scientific

For peer review only

Advisory Board. Statistics Finland approved access to records of deaths and sociodemographic data, and the National Institute for Health and Welfare (THL) for hospitalisation data of this sample.

The outputs of the research will include two papers: the mortality differences between survey participants and non-participants (step 1), and a comparison of the inference from the two methods (steps 2 and 3).

Beneficiaries and target audiences

Research on alcohol consumption, and more broadly, methods to improve on estimates derived from health surveys, will be of interest to a range of both academic and non-academic audiences including users of survey data, epidemiologists, public health and policy researchers, and governmental organisations. The findings of this validation exercise will have implications for general survey conduct: particularly if the methodology is shown to be invalid, consideration could be given to basing sampling frames on sources which readily identify non-participants as well as participants and enable linkage to administrative records at the individual level. Should the results of the future analysis demonstrate the validity of the methodology, the approach will be of benefit in the evaluation and creation of public health policy in both local and international governments.

Patient and Public Involvement

In this research data from general population, not on patients, was used. This analysis utilised two large pseudonymised record-linked administrative datasets with no possibility of direct participant contact beyond their initial participation in the Health 2000 study, due to data protection restrictions. Participants were not invited to contribute to the writing or editing of this document for readability or accuracy.

Data sharing statement

Access to the Health 2000 individual level survey responses can be requested via the Health 2000 homepage (thl.fi/health2000) from National Institute for Health and Welfare in Finland. Code used in the outputs will be made available in conjunction with their publication.

For peer review only

ADDITIONAL INFORMATION

Acknowledgements

We would like to thank the participants of the Health 2000 study, National Institute for Health and Welfare (THL) and Statistics Finland for the provision of the sociodemographic, hospitalisation and death data. Thanks in particular to Joonas J. J. Pitkänen from the Population Research Unit, Faculty of Social Sciences at the University of Helsinki for the preparation and provision of the population data.

Author Contributions

MAM prepared the first draft of this paper. The validation exercise was conceived by LG and AHL in discussion with PM. EG, TH and HR facilitated the acquisition of the survey and register-based data; PM facilitated the acquisition of the population sample data. HT contributed to the formulation of the approach. All authors contributed to all sections of the manuscript and approved the final version.

Funding

MAM, LG and AHL receive core funding from the Medical Research Council (MRC_MC_UU_12017/13) and the Scottish Government Chief Scientist Office (SPHSU13). TH, HR and HT are supported by the Academy of Finland under Grant 266251. PM is funded by the Academy of Finland.

Competing interests

MAM, PM, EG, HR, TH, HT, and LG have no conflicts to declare. AHL reports grants from Medical Research Council and Chief Scientist Office, during the conduct of the study.

Ethics approval

Research plans for this project have been approval by the Health 2000 Scientific Advisory Board. Access to register based sociodemographic variables and death data has been granted by Statistics Finland and to hospitalisation data by the National Institute for Health and Welfare (THL)

Figure Legends

Figure 1: Analytic sample selection process. Deaths and Hospitalisations to 31 December 2015.

Figure 2: Summary of proposed methodology

For peer review only

REFERENCES

1. Tolonen H, Helakorpi S, Talala K, et al. 25-year trends and socio-demographic differences in response rates: Finnish adult health behaviour survey. *Eur J Epidemiol* 2006;21(6):409-15.
2. Galea S, Tracy M. Participation Rates in Epidemiologic Studies. *Ann Epidemiol* 2007;17:643-53.
3. Mindell JS, Giampaoli S, Goesswald A, et al. Sample selection, recruitment and participation rates in health examination surveys in Europe – experience from seven national surveys. *BMC Med Res Methodol* 2015;15(1):78. doi: 10.1186/s12874-015-0072-4
4. Hara M, Sasaki S, Sobue T, et al. Comparison of cause-specific mortality between respondents and nonrespondents in a population-based prospective study. *J Clin Epidemiol* 2002;55(2):150-56. doi: 10.1016/S0895-4356(01)00431-0
5. Goldberg M, Chastang JF, Leclerc A, et al. Socioeconomic, demographic, occupational, and health factors associated with participation in a long-term epidemiologic survey: a prospective study of the French GAZEL cohort and its target population. *Am J Epidemiol* 2001;154(4):373-84. [published Online First: 2001/08/10]
6. Keyes KM, Rutherford C, Popham F, et al. How healthy are survey respondents compared with the general population? Using survey-linked death records to compare mortality outcomes. *Epidemiology* 2018;29(2):299-307. doi: 10.1097/ede.0000000000000775
7. Gorman E, Leyland AH, McCartney G, et al. Assessing the Representativeness of Population-Sampled Health Surveys Through Linkage to Administrative Data on Alcohol-Related Outcomes. *Am J Epidemiol* 2014;180(9):941-48. doi: 10.1093/aje/kwu207
8. Christensen AI, Ekholm O, Gray L, et al. What is wrong with non-respondents? Alcohol-, drug- and smoking-related mortality and morbidity in a 12-year follow-up study of respondents and non-respondents in the Danish Health and Morbidity Survey. *Addiction* 2015;110(9):1505-12. doi: 10.1111/add.12939
9. Jousilahti P, Salomaa V, Kuulasmaa K, et al. Total and cause specific mortality among participants and non-participants of population based health surveys: a comprehensive follow up of 54 372 Finnish men and women. *J Epidemiol Community Health* 2005;59(4):310-15. doi: 10.1136/jech.2004.024349
10. Gray L. The importance of post hoc approaches for overcoming non-response and attrition bias in population-sampled studies. *Soc Psychiatry Psychiatr Epidemiol* 2016;51(1):155-57. doi: 10.1007/s00127-015-1153-8
11. Gray L, McCartney G, White IR, et al. Use of record-linkage to handle non-response and improve alcohol consumption estimates in health survey data: a study protocol. *BMJ Open* 2013;3(3) doi: 10.1136/bmjopen-2013-002647
12. Gorman E, Leyland AH, McCartney G, et al. Adjustment for survey non-representativeness using record-linkage: refined estimates of alcohol consumption by deprivation in Scotland. *Addiction* 2017;112(7):1270-80. doi: 10.1111/add.13797
13. Gissler M, Haukka J. Finnish health and social welfare registers in epidemiological research. *Norsk Epidemiologi* 2004;14(1):113-20.
14. *Methodology Report: Health 2000 Survey*. Helsinki: KTL-National Public Health Institute, Finland Department of Health and Functional Capacity, 2008.
15. Health 2000. A Survey on Health and Functional Capacity in Finland: QUESTIONNAIRE 1, 2000. https://thl.fi/documents/189940/4108213/T2002_eng.pdf/83a57f85-0acb-4b25-af7d-29d7bf4b2282 (accessed 08/08/2018).
16. Hajat S, Haines A, Bulpitt C, et al. Patterns and determinants of alcohol consumption in people aged 75 years and older: results from the MRC trial of assessment and management of older people in the community. *Age Ageing* 2004;33(2):170-77. doi: 10.1093/ageing/afh046
17. Immonen S, Valvanne J, Pitkala KH. Prevalence of at-risk drinking among older adults and associated sociodemographic and health-related factors. *J Nutr Health Aging* 2011;15(9):789-94. [published Online First: 2011/11/18]

For peer review only

18. Niemelä H, Salminen K. Social security in Finland: Kela, Eläketurvakeskus, Työeläkevakuuttajat Tela ja sosiaali-ja terveysministeriö 2006.

19. Djerf K, Laiho J, Lehtonen R, et al. Weighting and Statistical Analysis. In: Heistaro S, ed. Methodology Report: Health 2000 Survey. Helsinki: KTL-National Public Health Institute, Finland Department of Health and Functional Capacity, 2008. http://urn.fi/URN:NBN:fi-fe201204193320.

20. Katikireddi SV, Whitley E, Lewsey J, et al. Socioeconomic status as an effect modifier of alcohol consumption and harm: analysis of linked cohort data. *The Lancet Public Health* 2017;2(6):e267-e76. doi: https://doi.org/10.1016/S2468-2667(17)30078-6

For peer review only

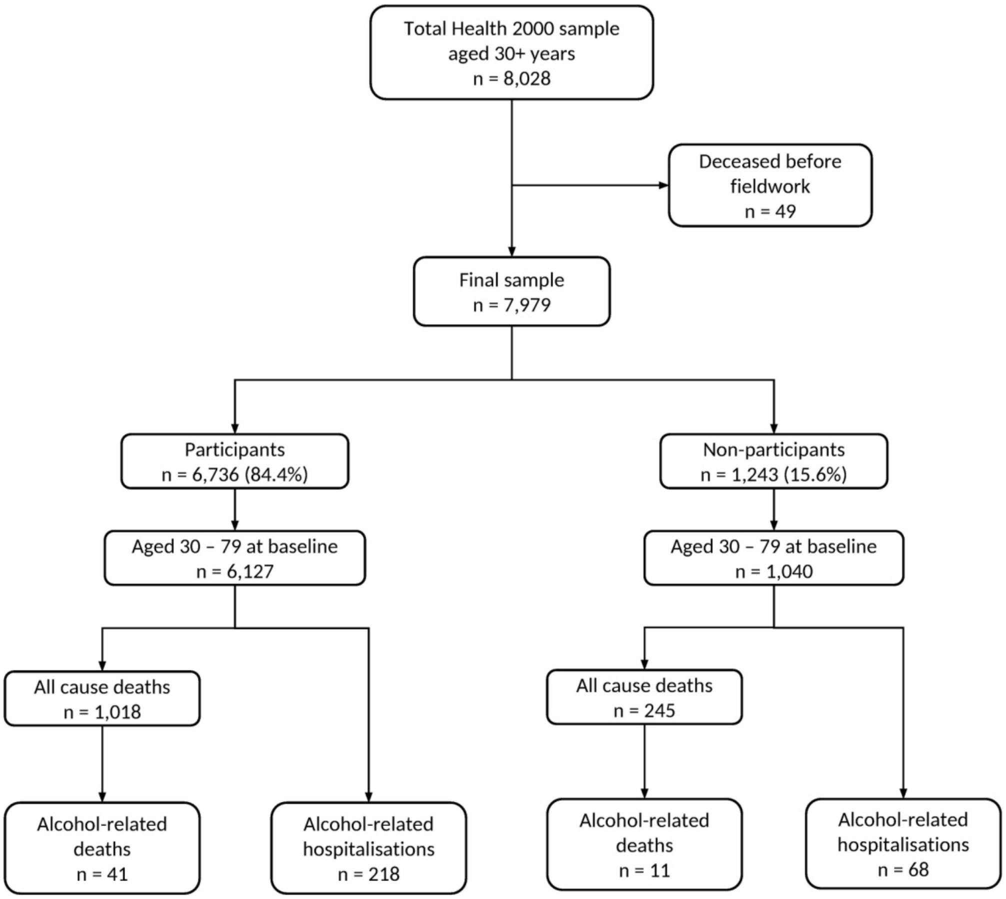

Analytic sample selection process. Deaths and Hospitalisations to 31 December 2015

150x134mm (300 x 300 DPI)

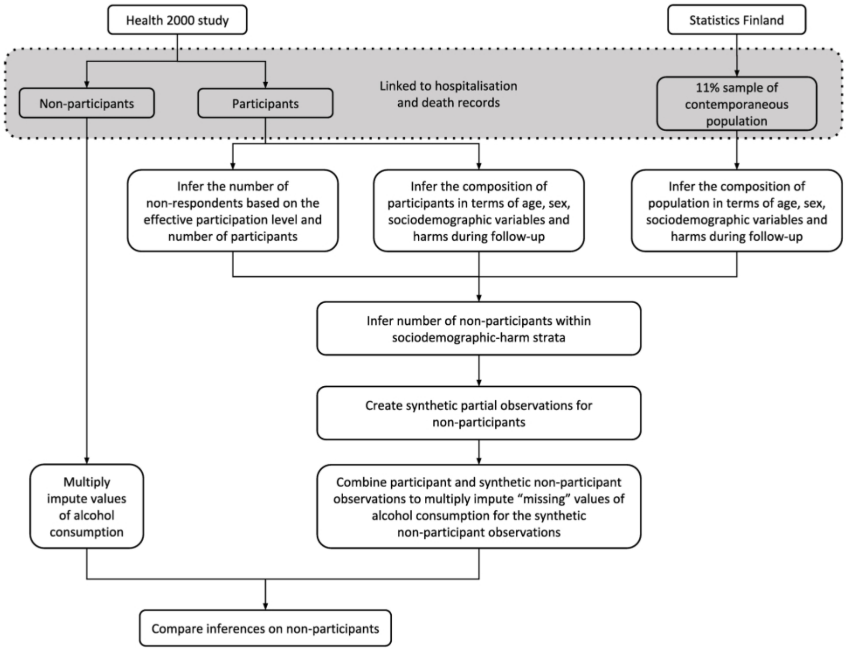

Summary of proposed methodology

190x142mm (300 x 300 DPI)
